# Results-based financing in health: from evidence to implementation

**DOI:** 10.2471/BLT.18.222968

**Published:** 2018-11-01

**Authors:** Michelle McIsaac, Joseph Kutzin, Elina Dale, Agnès Soucat

**Affiliations:** a Health Workforce Department, World Health Organization, avenue Appia 20, 1211 Geneva 27, Switzerland.; b Department of Health Systems Governance and Financing, World Health Organization, Geneva, Switzerland.

Results-based financing for health programmes are being piloted in many low- and middle-income countries. While the term results-based financing refers to demand- and supply-side incentives to increase output – that is, improved access to and quality of health care – this editorial focuses on the incentives that target service providers, also referred to as performance-based financing or pay-for-performance. Although the topic is well covered,^1^^–^^3^ the literature tends to examine pay-for-performance in isolation from the health system, rather focusing on the efficacy of the intervention.^4^ While useful, these studies provide insufficient guidance on how to scale up these interventions.

Unlike many pay-for-performance trials to date, except for a study in Rwanda,^5^ the pay-for-performance trial in Zambia^6^ published in this issue has three study groups: pay-for-performance, enhanced financing and business as usual. This division controls for the additional funding from the study.^6^ In Zambia, a large proportion of pay-for-performance project costs is for administration, including verification of outputs, with 47% of the budget for the pay-for-performance group allocated to incentive payments. The study concludes that the pay-for-performance intervention was cost–effective. However, cost–effectiveness is not the most interesting point of this study, as four policy relevant lessons emerge.

First, any output-based provider payment method requires some method of verification. If the pay-for-performance programme is scaled up in Zambia, the programme might transition to risk-based methods for verification, as in Zimbabwe,^7^ substantially bringing down the costs of verification. Verification costs associated with pay-for-performance implementation should be viewed in the context of provider payment methods and verification mechanisms already operational in the health system. In Zambia, setting up verification mechanisms required new investments,^8^ as before the pilot, providers were paid based on inputs. The estimates of the costs of the programme in Zambia, although annualized, are based on only 2.3 years of experience.^6^ Given that it is a new programme, one would expect that pay-for-performance verification costs would decline over time.

Second, approaching pay-for-performance as an either-or choice of financing is no longer the only frame of reference.². The substantive question is how to integrate elements of performance into the mixed provider payment system. There is an increasing consensus globally^9^ that countries need to move away from rigid input-based line-item budgets to mixed provider payment methods. How to use the pay-for-performance experience to shift to mixed provider payment methods is of more policy relevance than trying to assess whether pay-for-performance is more cost–effective than line-item payments. Research should therefore focus on those aspects of the pay-for-performance programmes that can be affordably integrated into health systems as a step towards more strategic purchasing.

Third, as described in the overall evaluation of the project,^8^ the direct disbursement of funds to facility bank accounts in the pay-for-performance group was a key ingredient for ensuring better service delivery. The enhanced financing group faced funds-flow issues, such as funds for health facilities not being disbursed in full.^8^ Some districts in the enhanced financing group used part of the project funds, which were meant for health facilities, for centralized procurements, and therefore facilities did not receive the full amount. 

Fourth, facility financial autonomy supported by pay-for-performance is key for ensuring progress towards strategic purchasing in Zambia. If balanced with clear accountability for both good results and the use of funds, it should be promoted further. In districts in the enhanced financing group, facilities only received in-kind goods and had little autonomy over spending. Funds flowed through district managers with rigid rules on spending, resulting in poor disbursement. On the contrary, while general spending parameters were given, facility managers in the pay-for-performance group could decide on how to spend funds.

In shifting towards mixed provider payment methods with timely disbursement of funds and greater financial autonomy by front-line providers, the budgeting processes need to be considered.^10^ In countries such as Zambia, where budgets are mainly formulated, approved and executed based on detailed input lines, shifting to payments based on performance could be challenging.^11^ Analysing the role of public financial management in health service delivery in countries like United Republic of Tanzania and Zambia is an important step in the right direction.^12^
